# Comparative analysis of percutaneous endoscopic interlaminar discectomy for highly downward-migrated disc herniation

**DOI:** 10.1186/s13018-023-04090-z

**Published:** 2023-08-14

**Authors:** Ran Li, Dongming Fu, Hao Han, Zihao Zhan, Yiang Wu, Bin Meng

**Affiliations:** Department of Orthopedics, The First Affiliated Hospital of Soochow University, Suzhou, Jiangsu China

**Keywords:** Percutaneous endoscopic interlaminar discectomy, Lumbar disc herniation, Migrated disc herniation, Complications

## Abstract

**Objectives:**

This study aimed to evaluate the clinical efficacy and safety of percutaneous endoscopic interlaminar discectomy (PEID) for treating highly downward-migrated disc herniation.

**Methods:**

We conducted a retrospective study on 39 patients with highly downward-migrated disc herniation who underwent PEID treatment between January 2015 and October 2020. The clinical outcomes, including the preoperative and postoperative visual analogue scale (VAS) for the back and leg, Oswestry Disability Index (ODI), and MacNab criteria for surgical success, were evaluated and compared to thirty-seven patients treated with posterior lumbar interbody fusion (PLIF).

**Results:**

The mean operation time of PEID was 93.00(77.00,110.00) min, while that of PLIF was 169.00(157.00,183.00) min. Continued improvement in both PEID and PLIF was observed in the VAS and ODI scores immediately after the surgery to the last follow‐up. The VAS and ODI scores of PEID one week after surgery were significantly different from those of PLIF. One patient with recurrent lumbar disc herniation in the same segment improved after undergoing repeat PEID, two patients had dura tears, and conservative treatment helped relieve the symptoms. The overall percentage of patients with good to excellent results of PELD according to the modified MacNab criteria was 97.43%, while that of PLIF was 94.60%.

**Conclusions:**

PEID has reliable efficacy and safety for treating highly downward-migrated disc herniation. And the long-term efficacy of PEID is comparable to PLIF. No severe complications occurred after surgery, and most patients’ symptoms were relieved.

## Introduction

Lumbar disc herniation is a series of diseases, including degeneration of the lumbar disc, rupture of the annulus fibrosus, protrusion of the nucleus pulposus tissue, irritation, and compression of nerve roots, resulting in back and leg pain, limb numbness, and other symptoms [1]. In migrated disc herniation, a large portion of the nucleus pulposus enters the spinal canal through the outlet of the severely ruptured annulus fibrosus, moves laterally, and further enters the dural sac, compressing the cauda equine [2]. Migrated disc herniation can be categorised as low grade, high grade, and very high grade [3]. When herniations extend beyond the height of the posterior disc margin, it is categorised as high grade. The nucleus pulposus enters the spinal canal through the posterior longitudinal ligament due to the complete rupture of the annulus fibrosus. The clinical symptoms are severe, because of which conservative treatment is ineffective, and surgical treatment is often required [4, 5]. Routinely, posterior open surgery is performed to resolve the problem. However, the incision is large, damaging the stable structure of the back of the spine, and postoperative complications such as low back pain occur easily [6, 7].

Percutaneous endoscopic lumbar discectomy (PELD) is a minimally invasive technique that can effectively replace traditional surgery for treating lumbar disc herniation [8, 9]. It can be divided into two approaches, which are as follows: Percutaneous endoscopic interlaminar discectomy (PEID) and percutaneous endoscopic transforaminal discectomy (PETD) [10]. Compared with traditional surgery, PELD maintains similar clinical efficacy and recurrence rate; however, it is less trauma, causes lesser bleeding, and has a lower incidence of surgical complications, a shorter operation time, a shorter hospital stay, and a faster recovery [11, 12]. Most types of intervertebral disc herniation, including an intervertebral disc protruding into the spinal canal, can be removed by PELD. However, PETD has certain limitations while treating highly migrated disc herniation. The failure rate of PETD while treating highly migrated intervertebral disc herniation increases due to anatomical obstruction and surgical instruments and techniques. PEID helps remove the disc directly by moving the working channel like a joystick. This study aims to analyse the follow‐up data of patients with highly downward-migrated disc herniation and explore the technical aspects of PEID and compare it with conventional open surgery posterior lumbar interbody fusion (PLIF), to obtain greater clinical efficacy and lower complications.

## Materials and methods

### General information

**Fig. 1:**
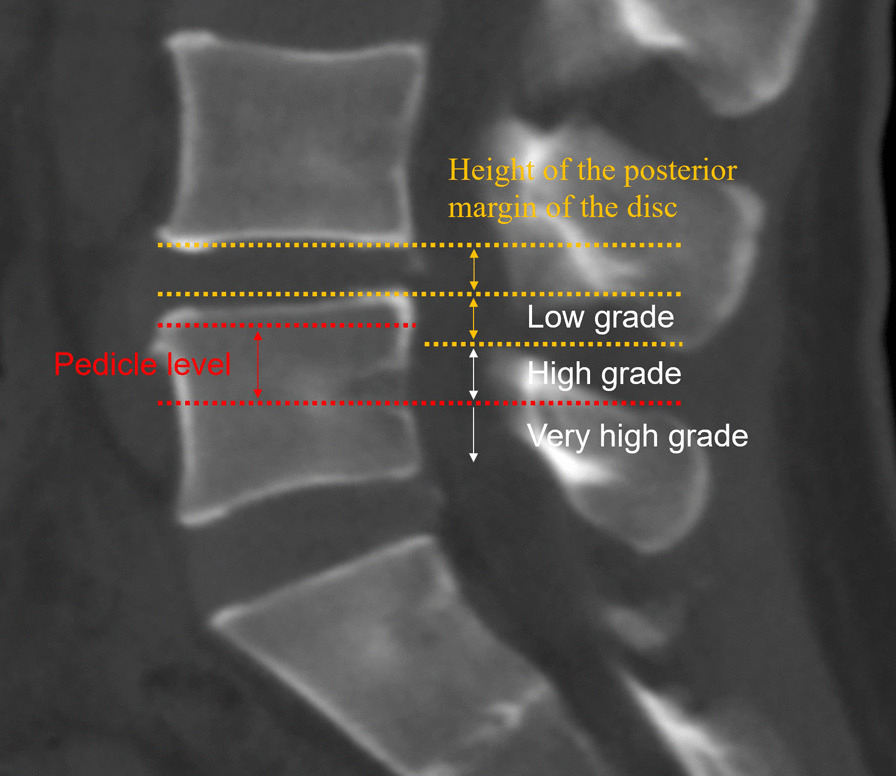
The extent of downward-migrated disc herniation. Low grade: Herniations within the height of the posterior disc margin; high grade: Herniations extending beyond the height of the posterior disc margin; very high grade: Herniations extending beyond the inferior margin of the pedicle

Patients with highly downward-migrated disc herniation who underwent PEID and PLIF were retrospective analysed and followed up from January 2015 to October 2020. The extent of downward-migrated disc herniation: Low grade: Herniations within the height of the posterior disc margin; high grade: Herniations extending beyond the height of the posterior disc margin; very high grade: Herniations extending beyond the inferior margin of the pedicle (Fig. 1) [13–15]. The following were the inclusion criteria: (1) Radiating pain and numbness in the unilateral lower extremity, (2) single-level highly downward-migrated lumbar disc herniation verified by imagological examination, (3) and conservative treatment, including bed rest, nonsteroidal anti-inflammatory drugs, and muscle relaxants, for at least 12 weeks, which had a minimal clinical signs and symptoms. The exclusion criteria were as follows: (1) Previous surgical history of treatment for spinal stenosis and motion instability, (2) other spinal disorders, such as ankylosing spondylitis and spinal tumours and fractures, and (3) dementia, intellectual disability, and drug abuse.

### Surgical methods

**Fig. 2:**
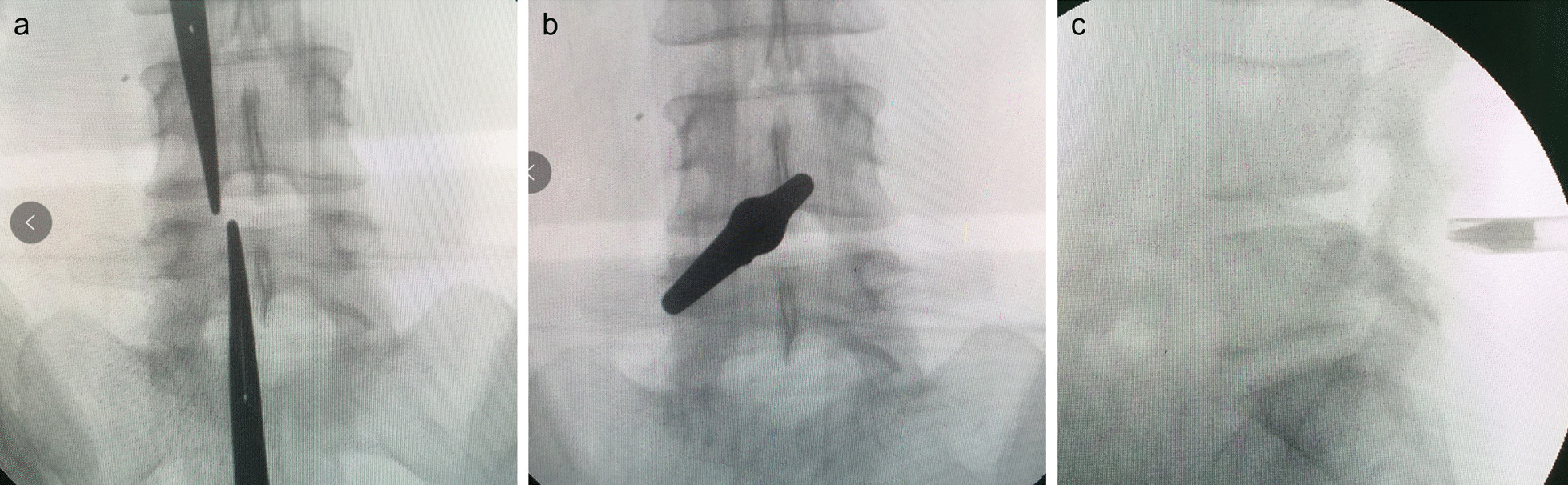
C-arm radiograph during surgery revealing the position of the sleeve. **a** C-arm was used to locate the lesion stage, and haemostatic forceps were used as reference; **b**, **c** Place the dilator

PEID: Four senior surgeons performed the surgery, following the same technical principles. All patients underwent the unilateral approach on the symptomatic side, under general anaesthesia. The patients were placed in the prone position on a Wilson table. An incision was made 1 cm lateral to the posterior midline on the level of the treated intervertebral space, with preoperative localisation as presented in Fig. 2. A dilator was bluntly inserted through the skin into the subcutaneous tissue and muscles. Once the dilator reached the ligamentum flavum, the working channel was inserted. The ligamentum flavum was dissected and separated, and epidural space was exposed. The herniated disc was removed using the straight and nucleus pulposus forceps. Adequate decompression was confirmed by probing the nerve roots. The tunnel was exited, and the wound was closed.

PLIF: The patients were placed in the prone position under general anaesthesia. The degenerated level was exposed from the standard middle incision. Standard decompression was performed in the segments after laminectomy. Adequate decompression was confirmed by probing the nerve roots. Next, extension of fixation and interbody fusion were performed. Suture the wound layer by layer.

Second-generation cephalosporins or clindamycin was prophylactically used during surgery. These medications were used after surgery only under special circumstances. In PEID group, all patients were encouraged to exercise with a lumbar brace on postoperative day 1 and were generally discharged on postoperative day 2, after which they were followed up at the outpatient clinic. A follow-up lumbar spine MRI is performed at one month after the surgery to assess the postoperative outcomes. In PLIF group, all patients were encouraged to exercise with a lumbar brace on postoperative day 3 and were generally discharged on postoperative day 4–5, after which they were followed up at the outpatient clinic.

### Clinical outcomes

The baseline characteristics and perioperative variables were recorded. Hospitalisation data were reviewed to identify any intraoperative complications. Adverse events occurring within 30 days after the surgery were defined as postoperative complications. The final outpatient appointment date at the Department of Orthopaedics was defined as the date of the last follow‐up visit. We also recorded and analysed the perioperative complications and their treatment. The extent of back pain and radicular leg pain was assessed using the visual analogue scale (VAS). Function and quality of life were evaluated using the Oswestry Disability Index (ODI). Data were recorded at five time points, which were as follows: Pre-operative, 1 week postoperatively, 1 month postoperatively, 3 months postoperatively, and the final follow‐up.

The surgical results were graded as “excellent” when there was no pain and no limitations for any activity, “good” when back or leg pain due to any strenuous activity was occasionally reported, “fair” when the symptoms improved after the surgery, but recurrent or residual pain led to restricted activities, and “poor” when the symptoms did not improve or worsened after the surgery.

### Statistical analysis

Normally distributed data are represented using the mean ± standard deviation (x̄ + s), and non-normally distributed data are represented using the median with the interquartile range (M (P25, P75)). We used the Kolmogorov–Smirnov test to determine, which variables were normally distributed. Student's t-test and ANOVA were used for parametric tests. Student–Newman–Keuls test and Pearson correlation analysis were used to calculate intergroup differences. For non-normally distributed data, Mann–Whitney's U-test, Kruskal–Wallis test, and Spearman's correlation analysis were used. Qualitative variables were compared using Chi-square test. Statistical significance was set at *p* < 0.05. All the data were analysed using SPSS 24.0 (SPSS Inc., Chicago, IL, USA).

## Results

### General information

**Fig. 3:**
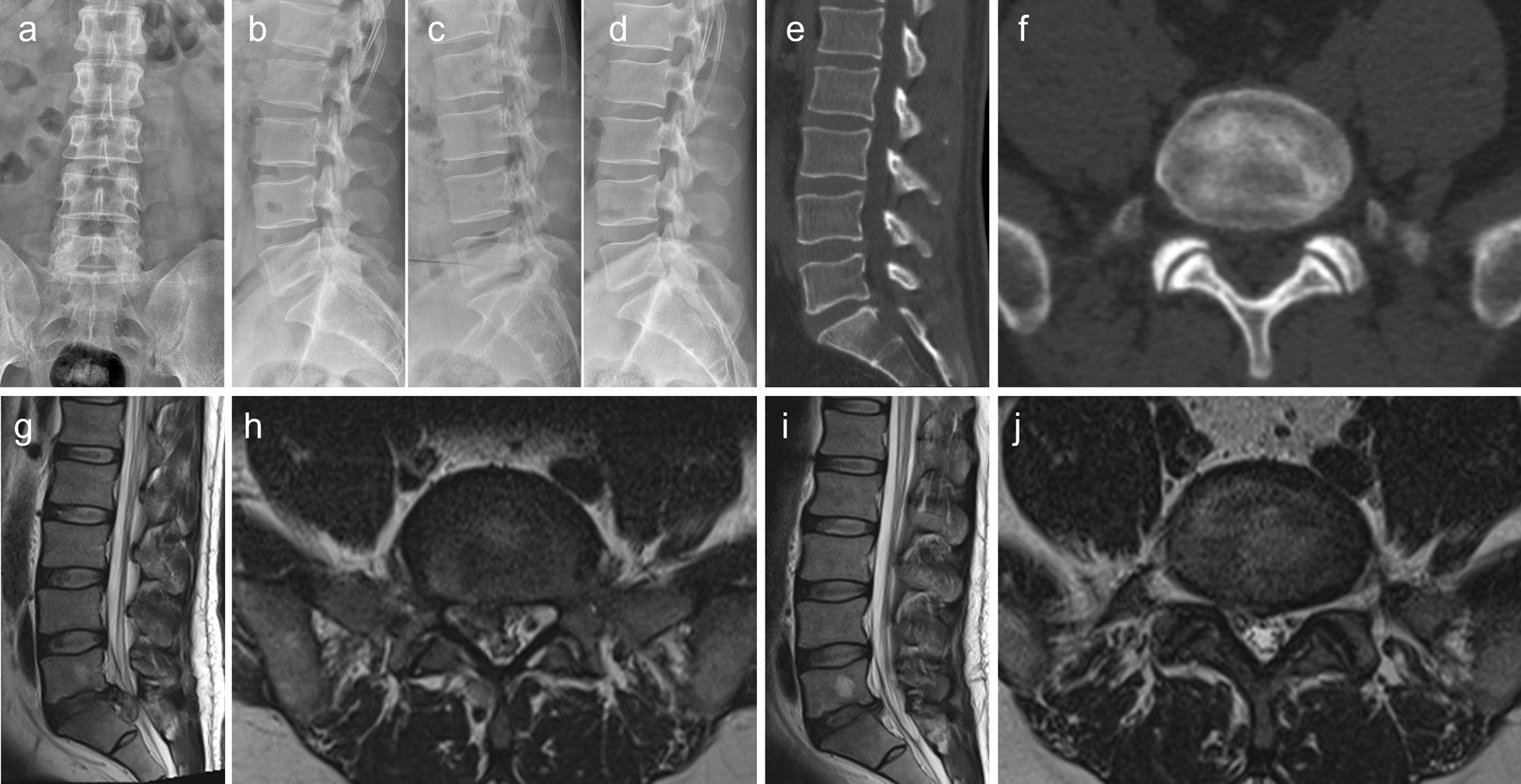
A 42-year-old man with highly downward-migrated disc herniation was treated with percutaneous endoscopic interlaminar discectomy. The patient mainly complained of low back pain with radiating pain in the right lower limb and cauda equina symptoms. The degree of prominence is very high grade. **a**, **b** Anterior and lateral lumbar radiographs; **c**, **d** Dynamic lumbar radiographs; **e**, **f** Computed tomography scan of the lower lumbar spine; **g**, **h** Magnetic resonance imaging (MRI) of the lumbar spine: Herniated disc at the L_5_-S_1_ segment, nucleus pulposus migration to the posterior edge of S_1_; **i**, **j** MRI revealing that the L_5_-S_1_ herniated disc and the free nucleus pulposus had been removed

**Fig. 4:**
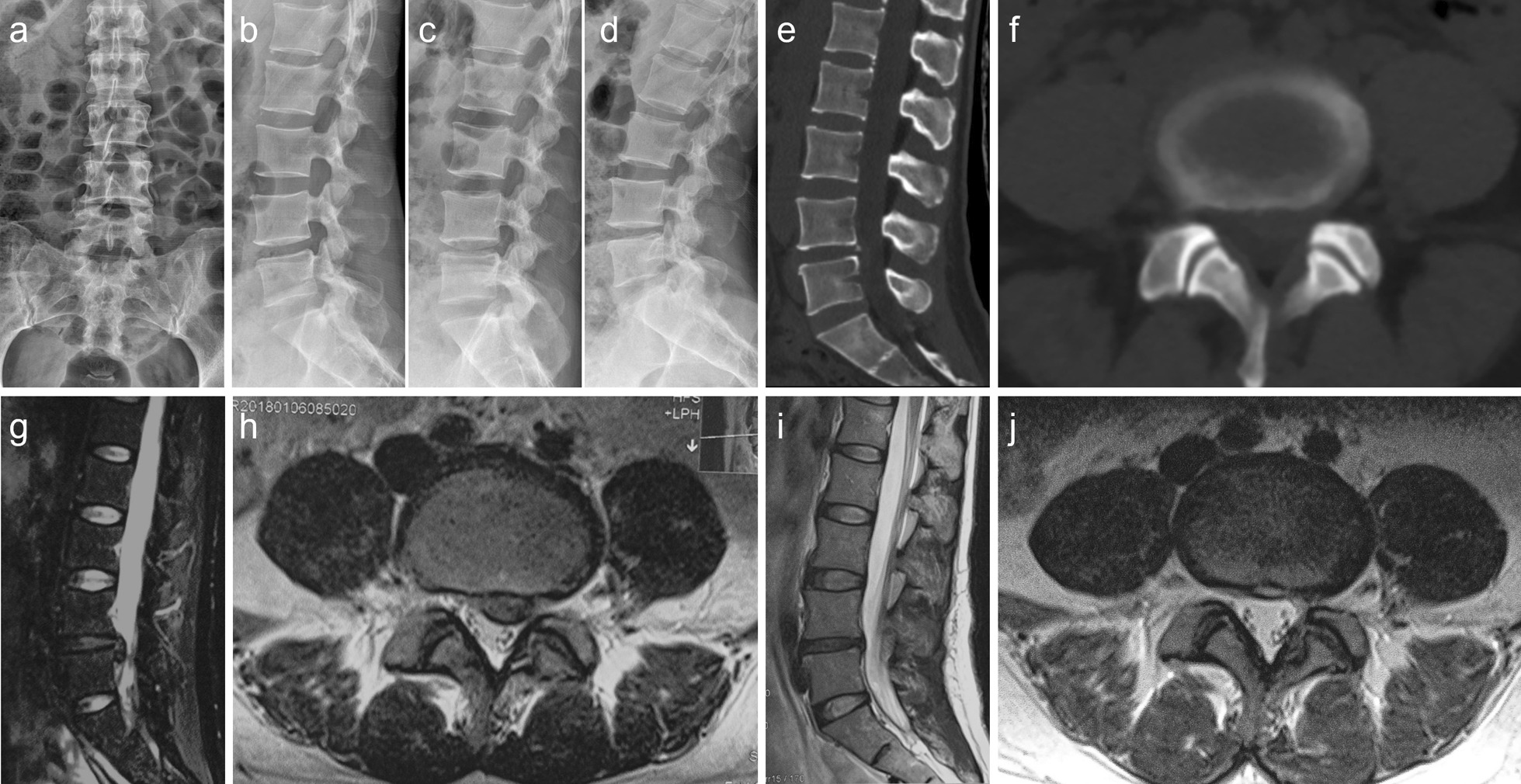
A 36-year-old female with highly downward-migrated disc herniation was treated with percutaneous endoscopic interlaminar discectomy. The patient mainly complained of radiating pain in the left lower limb. The degree of prominence is high grade. **a**, **b** Anterior and lateral lumbar radiographs; **c**, **d** Dynamic lumbar radiographs; **e**, **f** Computed tomography scan of the lower lumbar spine; **g**, **h** Magnetic resonance imaging (MRI) of the lumbar spine: Herniated disc at the L_4-5_ segment, nucleus pulposus migration to the posterior edge of L_5_; **i**, **j** MRI revealing that the L_4-5_ herniated disc and free nucleus pulposus had been removed

Thirty-nine patients after PEID and thirty-seven patients after PLIF met the inclusion criteria (Table 1). There were 21 males and 18 females in the PEID group. There were 19 males and 18 females in the PLIF group. The mean age of patients in the PEID group was 43.00(37.00,46.00) years, and the mean age of patients in the PLIF group was 44.00(43.00,45.00) years. There was no significant difference in age between the two groups. The mean follow-up time was 22(18,25) months in the PEID group and 25(20,29) month in the PLIF group. The mean operating time of the PEID group was 93.00(77.00,110.00) minutes, while that of the PLIF group was 169.00(157.00,183.00) minutes longer than that of the PEID group. No anaesthesia-related complications occurred in all patients. In PEID group, 15 cases of L_4-5_, and 24 cases of L_5_-S_1_ were compared, while in PLIF group, 13 cases and 24 cases were compared. The PEID group included 27 cases of high grade and 12 cases of very high grade, while the PLIF group included 27 cases of high grade and 10 cases of very high grade. The typical cases of PEID are presented in Figs. 3, 4.

**Table 1:** Demographics and surgical information

Variables		PEID	PLIF
Number		39	37
Sex (male/female)		21/18	19/18
Age (years)		43.00(37.00,46.00)	44.00(43.00,45.00)
Hospital time(days)		3.28 ± 0.97	7.18 ± 1.05
Intraoperative blood loss(ml)		Less	200.00(150.00,300.00)
Cost(yuan)		14,125.13 ± 750.56	26,530.63 ± 2785.37
Follow-up time (months)		28.00(18.00,36.00)	28.00(23.00,35.00)
Segments	L_4-5_	15	13
	L_5_-S_1_	24	24
Migration extent	High	27	27
	Very high	12	10
Operation duration (mins)		93.00(77.00,110.00)	169.00(157.00,183.00)
Resumption to duties(days)		22(18,25)	25(20,29)

### Clinical outcomes

The surgery was performed successfully performed in all patients, and they were followed up as mentioned in Table 2. The postoperative VAS and ODI scores significantly decreased compared with the preoperative scores (*p* < 0.05). The symptoms continued to improve at different time points after surgery in both groups. The mean VAS (low back) and VAS (leg) scores in PEID group improved from 6.97 ± 0.99 and 6.10 ± 0.79 preoperatively to 2.10 ± 0.88 and 1.46 ± 1.00 at 1 week postoperatively, which further increased to 2.00 ± 1.19 and 1.44 ± 0.60 at 3 months postoperatively, and at the final follow-up, the mean VAS (low back) and VAS (leg) scores were 2.36 ± 1.29 and 1.87 ± 1.15, respectively. The mean ODI scores improved from 70.00(66.00,74.00)% preoperatively to 16.00(14.00.22.00)% at 1 week postoperatively, which further increased to 20.00(16.00,24.00)% at 3 months postoperatively. At the final follow-up, the mean ODI score was 20.00(16.00,24.00)%. The mean VAS (low back) scores, mean VAS (leg) scores, and mean ODI scores were significantly higher in PLIF group than in PEID group at 1 week postoperatively. Otherwise, no significant differences were observed between the scores of the two groups. According to the modified MacNab criteria, 20 cases were categorised as excellent, 14 cases were categorised as good, and one case was categorised as fair, and the rate of excellent or good outcomes in PEID group was 97.43%. While the rate of excellent or good outcomes was 94.60% in PLIF group (Table 3). In the PEID group, the high and very high groups had similar clinical outcomes one week after surgery. The VAS (leg) in the high group was superior to that in the very high group at 1 month, 3 months, and the final follow-up (Table 4).

**Table 2:** Clinical outcomes before and after surgery

Group	Index	Preoperative	1 week	1 month	3 months	Final follow-up
PEID	VAS(low back)	6.97 ± 0.99	2.10 ± 0.88^a, b^	1.85 ± 0.90^a^	2.00 ± 1.19^a^	2.36 ± 1.29^a^
	VAS (leg)	6.10 ± 0.79	1.46 ± 1.00^a, b^	1.51 ± 0.60^a^	1.44 ± 0.60^a^	1.87 ± 1.15^a^
	ODI (%)	70.00(66.00,74.00)	16.00(14.00,22.00)^a, b^	18.00(16.00,22.00)^a^	20.00(16.00,24.00)^a^	20.00(16.00,24.00)^a^
PLIF	VAS(low back)	6.78 ± 0.78	3.08 ± 0.64^a, b^	1.65 ± 0.58^a^	1.59 ± 0.72^a^	1.92 ± 0.83^a^
	VAS (leg)	6.41 ± 0.72	2.43 ± 0.55^a, b^	1.38 ± 0.64^a^	1.43 ± 0.73^a^	1.57 ± 0.69^a^
	ODI (%)	72.00(68.00,75.00)	34.00(32.00,36.00)^a, b^	20.00(16.00,22.00)^a^	18.00(16.00,22.00)^a^	20.00(16.00,22.00)^a^

^a^*p* < 0.05 compared with preoperative data

^b^*p* < 0.05 compared with intergroup data

*VAS* visual analogue scale; *ODI* Oswestry Disability Index

**Table 3:** Modified MacNab criteria

Group	Index	Excellent	Good	Fair	Poor
PEID	Patients (*n*)	23	15	1	0
	Percentage (%)	58.97%	38.46%	2.57%	0
PLIF	Patients (*n*)	21	14	2	0
	Percentage (%)	56.76%	37.84%	5.40%	0

**Table 4:** Clinical outcomes of different degrees of lumbar disc herniation in PEID

Degree	Index	Preoperative	1 week	1 month	3 months	Final follow-up
High (*n* = 27)	VAS (low back)	6.93 ± 0.93	2.00 ± 0.94	1.82 ± 0.82	1.71 ± 1.39	2.25 ± 1.35
	VAS (leg)	6.04 ± 0.74	1.43 ± 0.84	1.39 ± 0.63*	1.43 ± 0.57*	1.57 ± 0.63*
	ODI (%)	68.00(64.00,72.00)	16.00(14.00,22.00)	18.00(14.00,22.00)	20.00(16.00,24.00)	18.00(14.00,24.00)
	MacNab	100%				
Very high (*n* = 12)	VAS (low back)	7.25 ± 1.06	2.33 ± 0.65	1.92 ± 1.08	2.67 ± 0.78*	2.58 ± 1.08
	VAS (leg)	6.25 ± 0.87	1.50 ± 1.31	1.83 ± 0.39*	2.08 ± 0.51*	2.33 ± 1.30*
	ODI (%)	71.00(70.00,74.00)	16.00(12.00,19.50)	18.00(16.00,21.50)	21.00(18.00,22.00)	24.00(18.50,27.50)*
	MacNab	91.67%				

^*^p < 0.05 compared with intergroup data

### Complications

At 6 months postoperatively, one patient had lumbar disc herniation at the same segment, which was successfully relieved via a second PEID. Two patients had mild dural tears causing numbness in both lower limbs after surgery, which gradually resolved after conservative treatment.

## Discussion

Migrated disc herniation is a unique type of lumbar disc herniation. It is characterised by degeneration resulting in a breach in the annulus fibrosus, and the nucleus pulposus extrudes and protrudes through the break, which is free under the posterior longitudinal ligament and can migrate to the cephalic and caudal side or even through the ligament into the spinal canal, compressing the nerve and resulting in symptoms [2]. After nucleus pulposus dissociation, in addition to mechanical compression, there are direct chemical stimulation occurs, resulting in congestion, oedema, adhesion, and nerve root degeneration. Therefore, patients often develop severe symptoms, severe pain, and symptoms such as numbness in the saddle area and difficulty in urination and defecation. Migrated disc herniation with progressive defects or cauda equina malformations, surgical treatment should be prepared as soon as possible to prevent irreversible damage caused by nerve root degeneration.

PELD has gradually become the preferred surgical technique for lumbar disc herniation as it is less traumatic compared with traditional open surgery, has a lesser impact on spinal stability, causes less intraoperative pulling of nerve roots and the dural sac, and is associated with a lesser risk of postoperative nerve damage [11, 12]. PELD has a relatively high failure rate while treating free disc herniations, particularly highly free disc herniations, due to its anatomical and operational limitations [16, 17]. While treating highly free intervertebral disc herniations via the transforaminal approach, the highly free nucleus pulposus might be obstructed by proliferative small joints, the pedicle of the lower vertebral body, and foraminal ligaments, which cannot be exposed under foraminal microscopy [17]. Meanwhile, due to the limited operation of the surgical channel system and the endoscope, flexibility during the surgery is limited, resulting in a high failure rate. However, PEID can be performed via the translaminar approach under the premise of preoperative positioning, and the endoscope can reach the head of the nucleus pulposus, thereby effectively lowering the difficulty and risk of nucleus pulposus removal [15]. During the surgery, the nucleus pulposus can be safely and completely removed under direct vision, with complete decompression, which effectively reduces the interference damage to the dural membrane and nerve roots and reduces the incidence of bleeding, nerve damage, and cerebrospinal fluid leakage. It is noteworthy that the free nucleus pulposus presses on the dural sac for a prolonged duration, with abundant blood vessels surrounding it, which could easily cause bleeding during surgery, thereby affecting the procedure [18]. Removal of the nucleus pulposus is particularly challenging in the case of highly free nucleus pulposus, thereby increasing the possibility of surgical failure.

PEID has a lower complication rate than traditional surgery, however, following an improper surgical technique is still likely to cause various complications, such as intervertebral space infection, residual nucleus pulposus, dural tear, nerve root injury, etc. [10, 11]. Herein, two patients had dura tears. Presumably, during the intraoperative removal of the nucleus pulposus tissue, the highly dissociated nucleus pulposus could compress the dura mater or even adhere to the dural sac, resulting in mechanical tear caused by instruments. We believe that adhesion is the main cause of endoscopic dural tear caused by large disc herniation. During the time of lumbar intervertebral disc herniation, long-term herniation will lead to adhesion between free nucleus pulposus and dura, which is also related to the location of free nucleus pulposus. As shown in the typical case in the article, as shown in Fig. 3, free nucleus pulposus is close to the spinal cord, and adhesion may occur when symptoms last for a long time. As shown in Fig. 4, the free nucleus pulposus is mainly located at the posterior edge of the vertebral body, so the possibility of adhesion is small. So, we think that when the nucleus pulposus is attached to the dura mater, the surgeon is trying to remove the free nucleus pulposus as much as possible, which can lead to a tear in the dura mater. Of course, it also depends on the skill and preparation of the surgeon. Therefore, in the early surgical stage, the surgeon should be cautious to prevent this complication while focusing on decompression. While treating highly downward-migrated disc herniation at L_3-4_ and higher levels through PEID, due to the small interlaminar window, several bony structures need to be removed, thereby decreasing the efficacy, and the nerve roots need to be pulled through the dorsal approach, making the intraoperative experience poor for patients under local anaesthesia [19]. One patient experienced recurrent lumbar intervertebral disc herniation at the operated level after engaging in manual labour for 6 months after surgery. The following factors need to be considered to achieve good outcomes: Familiarity with the PEID surgical technique; understanding the local anatomical structure of the lumbar spine and the compression factors associated with the patient’s case, along with detailed preoperative planning; gently performing the surgery under microscopy; the meninges and nerve roots in the compressed area should be carefully explored to avoid damaging them, the nucleus pulposus tissue should be removed completely, the decompression should be sufficient, the exploration should be repeated, and the preoperative images should be verified to avoid omissions.

This study had several limitations. This study was a retrospective analysis of medical records, and participants were operated on by multiple surgeons. The sample size of this study is small, the feasibility and benefits of PEID for high-grade lumbar disc herniation should be evaluated in a larger patient population with a prospectively controlled design.

## Conclusion

In summary, PEID effectively treats highly downward-migrated disc herniation and causes less trauma, brings about quicker recovery, is safe and effective, and has satisfactory short-term clinical efficacy. The effect of relieving pain at 1 month after PEID is better, and the long-term efficacy of PEID is comparable to PLIF. However, surgeons should grasp the indications, make detailed preoperative plans, and master the surgical technique.

## Data Availability

The data that support the findings of this study are included in this manuscript, and the original files are available from the corresponding author upon reasonable request.
